# Effectiveness of robot-assisted virtual reality mirror therapy for upper limb motor dysfunction after stroke: study protocol for a single-center randomized controlled clinical trial

**DOI:** 10.1186/s12883-022-02836-6

**Published:** 2022-08-22

**Authors:** Dong Wei, Xu-Yun Hua, Mou-Xiong Zheng, Jia-Jia Wu, Jian-Guang Xu

**Affiliations:** 1 Shanghai University of Traditional Chinese Medicine, Shanghai, 201203 China; 2 Shanghai University of Traditional Chinese Medicine Yueyang Hospital of Integrated Traditional Chinese Medicine and Western Medicine, Shanghai, 200437 China

**Keywords:** Upper limb motor dysfunction, Stroke, Robot-assisted virtual reality mirror therapy (RAVRMT), Randomized controlled trial, Functional magnetic resonance imaging (fMRI)

## Abstract

**Background:**

Upper limb motor dysfunction is a common sequela of stroke, and its clinical efficacy needs to be improved. This protocol describes a trial to verify the clinical efficacy of robot-assisted virtual reality mirror therapy (RAVRMT) in improving upper limb motor dysfunction in stroke patients, and to explore the central mechanism by using functional magnetic resonance imaging (fMRI).

**Methods:**

This trial will be a single-center, assessor-blinded, randomized controlled clinical study. Thirty-two eligible patients will be randomly divided into 2 groups according to the ratio of 1:1, namely virtual reality mirror therapy (VRMT) group and robot-assisted virtual reality mirror therapy (RAVRMT) group. The interventions will be performed once a day for 4 weeks. Primary outcome is Fugl–Meyer motor function assessment-Upper Extremity (FMA-UE), secondary outcomes are the Montreal Cognitive Assessment (MoCA), activities of daily living (ADL), quality of life (QOL), the pain visual analogue scale (VAS-pain) and fMRI. Adverse events will be recorded, and severe adverse events will be used as criteria to discontinue the intervention.

**Discussion:**

Combined application of robot-assisted therapy and virtual reality mirror therapy could theoretically activate mirror neuron system and reward circuits to a greater extent, but further high-quality research is needed. The results of this trial will determine whether RAVRMT could better improve upper limb motor dysfunction after stroke and explore its central mechanism using fMRI.

**Trial registration:**

This trial was prospectively registered at ClinicalTrials.gov (ChiCTR2200061721; 01 July 2022).

## Background

In recent years, stroke has become the second leading cause of death and the third leading cause of disability in the world [1]. Studies have shown that about 80% of acute stroke patients and more than 55% chronic stroke patients have upper limb motor dysfunction [2, 3], which seriously affects the quality of life of patients and their families [4, 5]. At present, the conventional rehabilitation methods for upper limb motor dysfunction after stroke consists of physical therapy [6], occupational therapy [7], but more than 50% of stroke patients still have long-term upper limb motor dysfunction [8]. The clinical efficacy of conventional rehabilitation methods in the treatment of upper limb motor dysfunction after stroke is not satisfactory, and new rehabilitation concepts and techniques are urgently needed.

How can we better promote the recovery of upper limb motor dysfunction after stroke? The disruption of motor-related brain networks is the primary cause of upper limb motor dysfunction after stroke, and another important cause is the upper limb disuse caused by long-term inactivity, so a single treatment for one cause is not adequate [9]. In stroke, the disruption of motor-related brain networks is the damage at the central level [10] and the upper limb disuse caused by long-term inactivity is the damage at the peripheral level [11]. As a clinical central intervention technology, mirror therapy can activate the motor-related brain network through action observation [12]. A recent meta-analysis demonstrates that mirror therapy can significantly improve upper limb motor dysfunction in stroke patients [13]. As a new kind of mirror therapy, virtual reality mirror therapy (VRMT) can provide immersion mirror therapy for stroke patients [14]. However, mirror therapy lacks the participation of proprioception [15], which may not be able to simulate the actual scene well and activate the motor-related brain network to the greatest extent. As a clinical peripheral intervention technology, a large number of studies have proved that robot-assisted therapy (RAT) can have a positive impact on the recovery of upper limb motor dysfunction in stroke patients through programmed task training [16, 17], including motor function and proprioception. At present, more and more studies show that mirror therapy combined with other interventions is superior to a single rehabilitation therapy in the treatment of upper limb motor dysfunction in stroke patients [18–20].

In this study, we designed a device that combined RAT and immersion VRMT to treat upper limb motor dysfunction after stroke. Moreover, the device can dynamically match the motion trajectory of the RAT with the motion images displayed in real time by the VRMT, so as to realize the cycle of “visual information input-visual information output-motion feedback” in the stroke patient. This is different from other combinations of a central intervention and a peripheral intervention for upper limb motor dysfunction after stroke [21, 22], which a central intervention and a peripheral intervention do not dynamically match each other in real time. In summary, the device is an innovative medical rehabilitation machine, which realizes the cycle of “visual information input-visual information output-motion feedback”, and provides patients with a full-body immersive experience that is consistent with vision, touch, and proprioception. We call the treatment robot-assisted virtual reality mirror therapy (RAVRMT) and the device RAVRMT robot. The RAVRMT robot is ultimately expected to activate mirror neuron system (MNS) and reward circuits to the greatest extent possible, thereby promoting the improvement of upper limb motor dysfunction in patients after stroke.

Therefore, we designed a randomized controlled trial to explore the clinical efficacy of RAVRMT in improving upper limb motor dysfunction in patients after stroke. Meanwhile, in this trial, functional magnetic resonance imaging (fMRI) scans will be performed to find significant changes in motor-related brain functional connectivity, and the motor-related brain functional network will be compared before and after the intervention to further reveal the brain neural mechanism of RAVRMT’s effects.

## Methods and design

### Study design

**Fig. 1:**
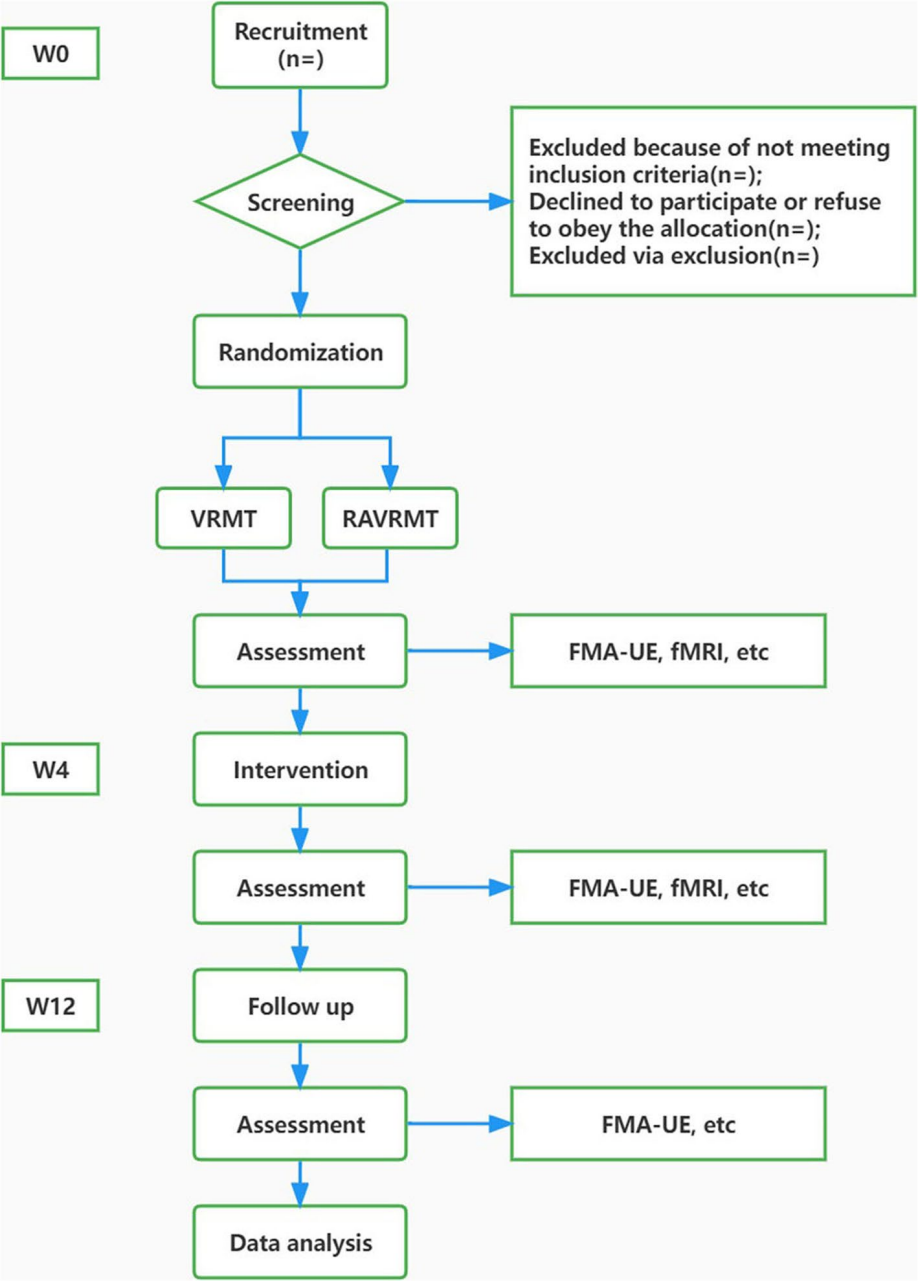
Flowchart of study design. W, week

This is a single-center, assessor-blinded, randomized controlled clinical trial. This trial was approved by the Ethics Committee of Yueyang Hospital of Integrated Traditional Chinese Medicine and Western Medicine. In this trial, a randomized controlled study will be used, and all eligible patients will be randomly divided into 2 groups according to the ratio of 1:1, namely VRMT group and RAVRMT group. There will be a 4-week intervention and a 12-week follow-up. Outcome measurements include the Fugl-Meyer motor function assessment -Upper Extremity (FMA-UE), fMRI, and others. The flowchart of this trial is shown in Fig. 1.

### Recruitment

Patients will be recruited from the Yueyang Hospital of Integrated Traditional Chinese Medicine and Western Medicine. The study started in June 2022; the end date is planned in June 2023 (Chinese Clinical Trial Registry, ChiCTR2200061721). We advertise the study online (website) and offline (poster) to recruit patients. To enhance recruitment quality, investigators monitor medical records. The attending doctor will carefully inquire and record the patients’ past and present medical history.

### Inclusion criteria

The Inclusion criteria include the following: (1) Diagnosis of stroke by clinical assessment and neuroimaging as defined by the International Classification of Diseases (ICD-11); (2) Regardless of gender, age between 30 and 70; (3) Hemiplegic; (4) Primary stroke, stroke course of 1–6 months; (5) Primary school or above, with normal cognitive function (Montreal Cognitive Assessment (MoCA) score of 26 or above); (6) Sufficient audiovisual level to complete necessary research examinations and virtual reality mirror therapy; (7) Patient’s informed consent.

### Exclusion criteria

The exclusion criteria include the following: (1) Severe comorbidities that prevents patients from cooperating with treatment, such as severe heart disease, severe bleeding, coagulation disorders, etc.; (2) Severe spasticity of the affected upper limb; (3) Skin damage of the affected upper limb; (4) History of mental illness; (5) Pregnant and lactating women.

### Study setting

**Fig. 2:**
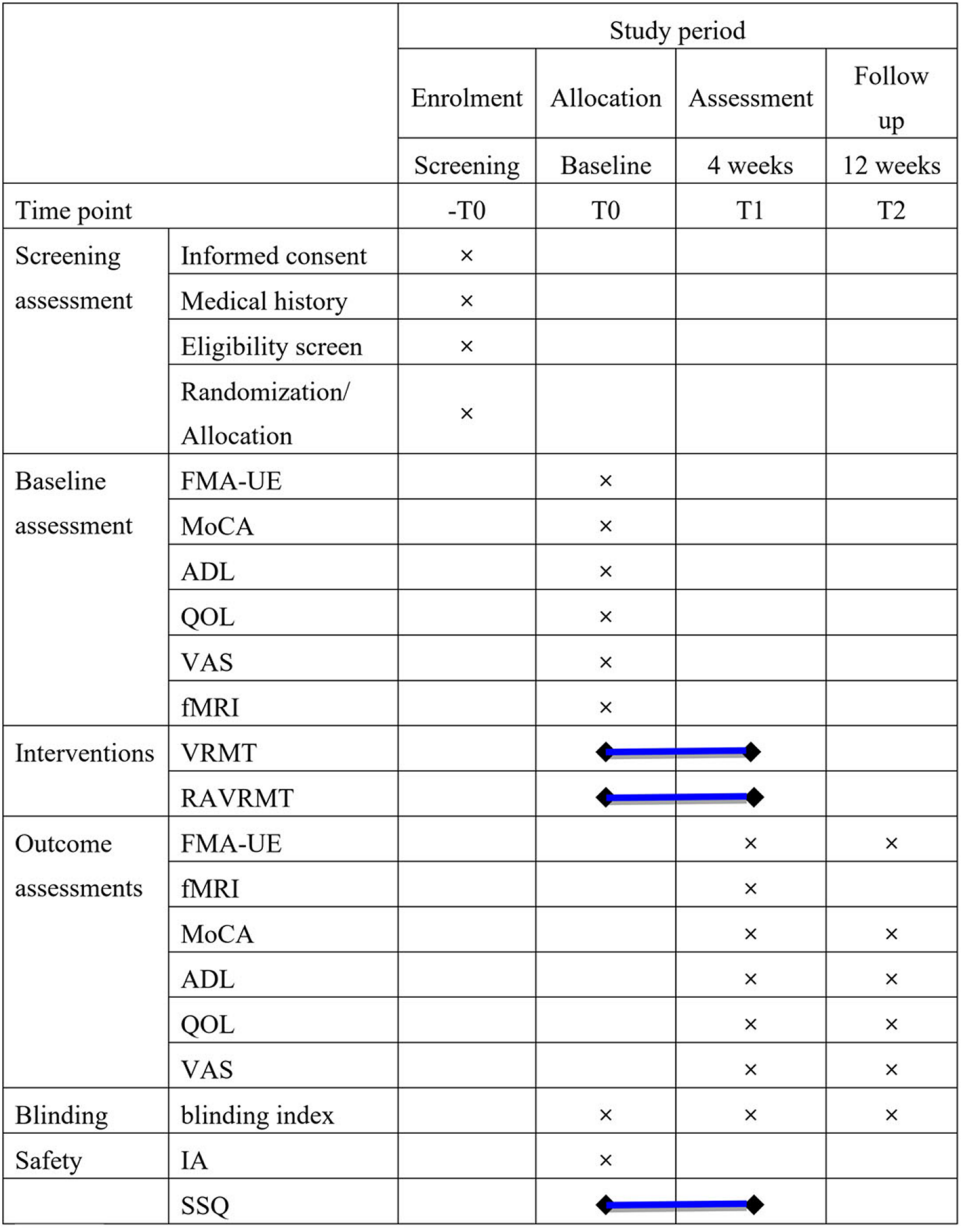
The SPIRIT schedule of enrolment, interventions, and assessments

The treatments will be carried out in Yueyang Hospital of Integrated Traditional Chinese Medicine and Western Medicine. Patients participating in the study are required to sign an informed consent form and voluntarily participate in the study. Primary outcome is FMA-UE, secondary outcomes are the Montreal Cognitive Assessment (MoCA), activities of daily living (ADL), quality of life (QOL), the pain visual analogue scale (VAS-pain) and fMRI. The trial (SPIRIT) schedule for patient enrolment, interventions, and assessments is shown in Fig. 2.

### Sample size

The sample size was calculated based on the main efficacy indicator (FMA-UE score). We referred to a previous similar study as the basis for estimating the sample size [23]. The means and standard deviations (SDs) of FMA-UE scores in the two groups were 30.41 ± 9.07, 23.00 ± 5.58. The sample size estimation formula is as follows:

$$ n=\frac{{\left({Z}_{\upalpha}+{Z}_{\beta}\right)}^2{\sigma}^2}{{\left({u}_r-{u}_c-\varDelta \right)}^2} $$

In this formula, *n* is the required number of each group; Assuming one-sided *α* = 0.025 and one-sided *β* = 0.1; The value of Δ is 10% of the mean of the control group in the previous similar study [24]; The required sample size is 13 in each group; Considering a 20% dropout rate, a total of 32 participants should be recruited (16 per group).

### Randomization and allocation

Randomization is conducted by an independent investigator who is not involved in the recruitment, assessment, treatment, or data analysis. The randomization sequence is generated by computer-generated random numbers. Thirty-two eligible patients are randomly divided into VRMT group and RAVRMT group in a ratio of 1:1. The assignment of patients is concealed in an opaque, sealed envelope, and cases are numbered by an independent investigator in the order of entry into the group. Before starting an intervention, inform therapists of the patient’s specific number and intervention information in order to obtain the assigned treatment.

### Blinding

In this study, an assessor-blind method will be used. The assessor will evaluate the study results without knowing patients’ assignment, and conclusions will be drawn by a statistical analyst who is not involved in recruitment, screening, assessment, or intervention. In addition, the treatment process is performed independently by therapists who will not be involved in the procedures for information collection, assessment and data analysis. To minimize bias and interference, therapists do not provide patients with any information about the benefits and risks of treatment. The results of the assessment will be kept confidential to the researchers to ensure the objectivity of the assignment and the reliability of the study results. An independent staff will supervise the implementation of the blind method, and the blind code will be made public after completion of the statistical analysis. In the event of blinding failure, the patient will be excluded from the study protocol.

### Interventions

#### Groups

VRMT group: conventional therapy + VRMT;

RAVRMT group: conventional therapy + RAVRMT.

The interventions will be performed once a day for 4 weeks. VRMT and RAVRMT will be performed for 20 min per day. Follow-up will follow 12 weeks later.

#### Conventional therapy

All patients will receive conventional rehabilitation therapy provided by the Yueyang Hospital of Traditional Chinese Medicine and Western Medicine according to the results of rehabilitation assessment. Conventional rehabilitation therapy includes acupuncture, massage, physical factor therapy, exercise therapy such as posture and good limb position, joint stretch training, neurodevelopmental therapy (PNF technique, Bobath technique, Brunnstrom technique and Rood technique), active or passive limb training, etc. The conventional therapy received will be recorded but not influenced. At the same time, doctors will make basic medication plans according to the specific conditions of individual patients. We will track and record the type, frequency and dose of patients’ medication.

### Virtual reality mirror therapy

**Fig. 3:**
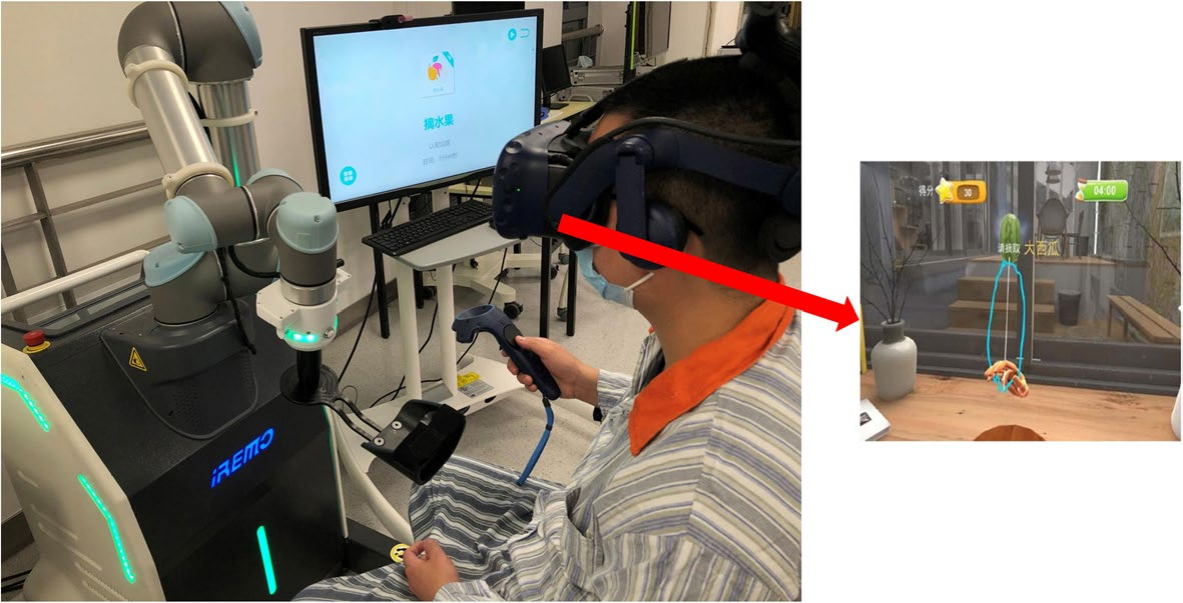
Schematic diagram of virtual reality mirror therapy

The VRMT group will receive VRMT using a clinically evaluated medical device (the RAVRMT robot). First, an occupational therapist with skilled operation explains the definition and content of VRMT to the patient. Second, the patient sits in front of the device, with a virtual reality head-mounted display on the patient’s head. Then, the unaffected hand holds the control handle to perform various motion trajectories in the game, which are transferred to the computer system and mirrored in real time on the virtual reality head-mounted display to show the various motion trajectories of the affected hand. Finally, the patient is required to carefully observe various dynamic simulation images of the affected hand through the virtual reality head-mounted display (Fig. 3).

### Robot-assisted virtual reality mirror therapy

**Fig. 4:**
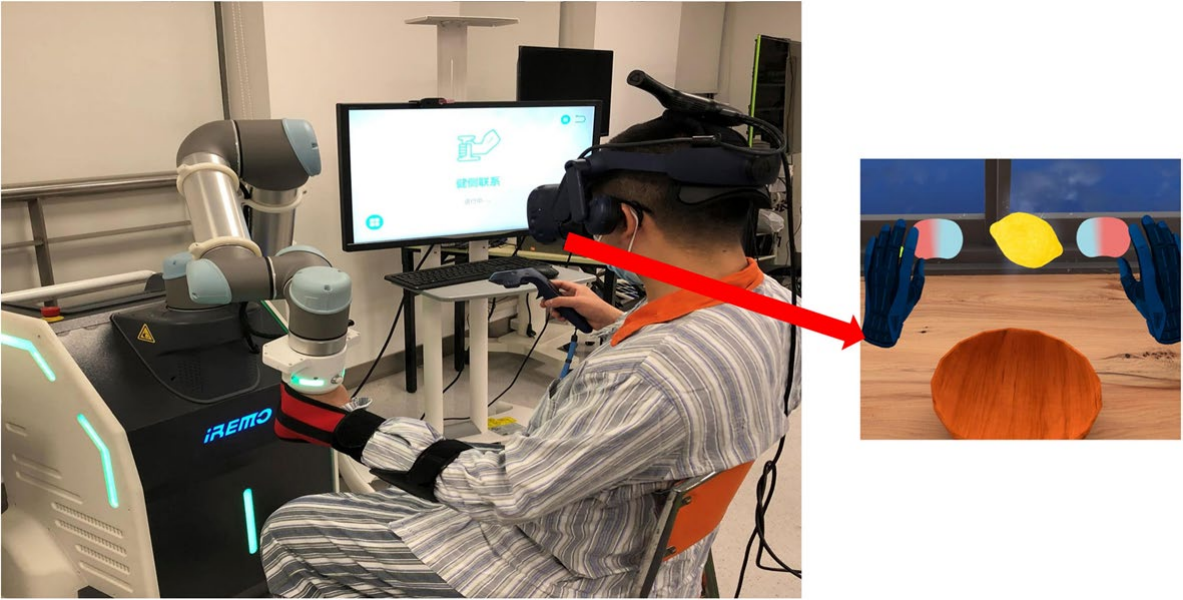
Schematic diagram of robot-assisted virtual reality mirror therapy. The patient’s left upper limb is on the affected side, and the right hand holds the control handle

The RAVRMT group will receive RAVRMT using a clinically evaluated medical device (the RAVRMT robot). First, an occupational therapist with skilled operation explains the definition and content of RAVRMT to the patient. Second, the patient sits in front of the device, the affected upper limb is attached to the traction end of the device, the unaffected upper limb holds a control handle. Third, the unaffected hand holds the control handle to perform various motion trajectories, these trajectories are transferred to the computer system, and real-time simulations display on a virtual reality head-mounted display. Then, the traction end of the device guides the affected upper limb to perform these same motion trajectories, and real-time simulations display on the virtual reality head-mounted display. Finally, the unaffected hand holding the control handle and the affected upper limb guided by the traction end of the device move together to complete the game in virtual reality (Fig. 4).

### Follow up

To further explore the long-term effects of this trial, follow-up of patients after the 4-week intervention is required. All patients will participate in an 8-week unsupervised follow-up period immediately after the end of a 4-week intervention. At week 8 of the end of 4-week intervention, patients will undergo clinical assessments to assess their FMA-UE, etc.

### Assessment

The study period for each patient is 12 weeks. All patients will be assessed before and after a 4-week intervention for FMA-UE, MoCA, ADL, QOL, VAS, fMRI and safety measures. At week 8 after the end of 4-week intervention, all patients will be assessed their FMA-UE, MoCA, ADL, QOL, and VAS. All outcomes will be measured by assessors who have been uniformly trained, blinded to the randomization group after the baseline visit assessment.

### Primary outcome

The primary outcome of this study is the mean change in FMA-UE scores between and within the two groups before and after the intervention and follow-up. FMA-UE is widely used in the assessment of upper limb motor function and can reflect the level of upper limb motor function in stroke patients. The total score of the upper limb is 66 points, which is divided into three parts: shoulder–arm (36 points), wrist–hand (24 points), and coordination (6 points), and lower scores indicate worse upper limb movement function. The FMA-UE is often used as the gold standard to test the validity of other scales [9, 25]. FMA-UE has excellent reliability and validity and is sufficiently sensitive to clinical and research practice [20].

### Secondary outcomes

The Montreal Cognitive Assessment (MoCA) scale is an assessment tool for rapid screening for mild Cognitive impairment. The Cognitive areas assessed include attention and concentration, executive function, memory, language, visual structural skills, abstract thinking, and computational and directional abilities. The total score of the scale is 30, and the test result shows that the normal value is ≥26.

Patient’s activity of daily living (ADL) is assessed by using the Modified Barthel index (BI), including the patient’s stool, urination, grooming, toileting, eating, transfer, walking, dressing, going up and down stairs, and bathing. A normal score is 100, representing self-care. ADL can be divided into mild (61–99 points), moderate (41–60 points), severe (1–40 points), and completely dependent (0 points).

Quality of Life (QOL) is assessed by using the Short Form 36-Item Health Survey (SF-36), including 8 dimensions: physical functioning, physical and emotional role limitations, bodily pain, social functioning, mental health, vitality, and general health perceptions. SF-36 assesses the quality of life of the disabled older adults, and has proven to have good reliability and validity [26].

The pain Visual Analogue Scale (VAS) is widely used in medicine to measure pain intensity [27]. Draw a 10 cm horizontal line on the paper. One end of the horizontal line is 0, which means no pain; The other end is 10, which means severe pain; The middle section shows different levels of pain. The patient is asked to mark the level of pain on a horizontal line according to how they feel.

fMRI is a non-invasive imaging technique that uses magnetic resonance to measure changes in brain hemodynamics caused by neuronal activity to reflect the activation state of brain regions. The fMRI scans of this study are acquired on a 3-Tesla scanner (SIEMENS VERIO, Erlangen, Germany) in the Yueyang Hospital of Integrated Traditional Chinese Medicine and Western Medicine. In the study, we acquire resting-state fMRI and T1-weighted image scans. Patients will be asked to lay supine in the scanner with their eyes closed but remain awake, and their heads are immobilized with foam pads to minimize head motion. The resting-state fMRI are acquired with the following parameters: repetition time (TR) = 2000 ms, echo time (TE) = 30 ms, flip angle (FA) = 90°, field of view (FOV) = 256 mm*256 mm, matrix = 256*256, slice thickness = 3 mm (no gap) and number of slices = 33. The 3D T1-weighted images are acquired using a brain volume sequence with the following parameters: TR = 1900 ms, TE = 4.6 ms, FA = 9°, FOV = 256 mm*256 mm, matrix = 256*256, slice thickness = 1 mm (no gap) and the number of slices = 160. After data preprocessing, the following indicators can be obtained: Amplitude of Low Frequency Fluctuation (ALFF), regional homogeneity (ReHo), Functional Connectivity (FC), Independent Component Analysis (ICA). ALFF is an effective method to reflect local brain activity by detecting low frequency (0.01–0.1 Hz) fluctuations of the blood oxygen level dependent (BOLD) signal [28]. ReHo reflects the correlation between the time series of a given voxel and that of its neighbors [29]. FC reflects the correlation between different brain regions [30]. ICA is an algorithm of multiple regression analysis, which distinguishes the components of the brain’s resting state network from one of the confounding components [31].

### Safety outcomes

We assess the acceptability and safety of the therapy. Before each patient will be treated, the Internet Addiction (IA) Scale will be used to measure the level of Internet addiction [32]. The Internet Addiction Scale includes: compulsive use, withdrawal, tolerance, time management problems, and interpersonal and health problems. Adverse events such as headaches, dizziness, nausea, vomit, fatigue, or epileptic seizures will be considered safety outcomes. Before and after each treatment, patients will be asked about their physical condition and whether they feel uncomfortable. For each patient, adverse events will be assessed by the simulator sickness question (SSQ) before and after each treatment. The SSQ scale contains 16 items, for example, sweating, nausea, dizziness, burping, headache, blurred vision, and feeling dizzy [33]. Participants will choose “None,” “slight,” “Moderate” or “severe” according to the severity of each item. It is a gold standard for assessing physical illness after exposure to VR environments. Adverse events will be recorded, and severe adverse events will be used as criteria to discontinue the intervention.

An independent safety staff will collect, evaluate, report all adverse events and report directly to the Ethics Committee of Yueyang Hospital of Integrated Traditional Chinese Medicine and Western Medicine.

### Data management

The data will be stored both in hard copy and in digital version as well.

### Statistical analysis

Blind the statistician. The clinical data will be analyzed by SPSS25.0 software. For the measurement data of the obtained data, the normality test is first performed. For the measurement data conforming to the normal distribution, it is expressed as the mean ± standard deviation (x ± s). The paired t test is performed before and after the two groups, and the comparison between the two groups is used Two-way repeated measures analysis of variance (2-way repeated measures ANOVA) test. The non-normally distributed measurement data before and after a group of self-comparison uses the Wilcoxon signed rank test, and comparison between the two groups uses the Mann-Whitney signed rank test. The significance level of the statistical test is set to α = 0.05, and the one-sided test is performed. A data monitoring committee will not be implemented because all participants will be treated and adverse events are expected to be low.

## Discussion

Mirror therapy is based on the theory of mirror neuron system (MNS) and has been widely used in post-stroke rehabilitation [34]. The MNS refers to the brain regions that activate when an individual performs a specific action and when others are observed performing the same or similar action [35]. The human MNS includes the bilateral posterior inferior frontal gyrus (Broca’s area on the left), the ventral premotor cortex, the inferior parietal lobule (angular/supramarginal gyrus), the posterior superior temporal gyrus (Wernicke’s area on the left) [34]. The bilateral posterior inferior frontal gyrus, the ventral premotor cortex, the inferior parietal lobule are all brain regions involved in motor execution [36]. Mirror therapy can improve upper limb motor dysfunction in stroke patients by activating MNS [12, 13]. Virtual reality is used to improve the “immersion” of patients in the process of mirror therapy and reduce the influence of external interference factors [14].

Robot-assisted therapy (RAT) can provide programmed upper limb task training to simulate normal upper limb functional movements. Robot-assisted programmed task training activates cortical striatum pathways that promote motor control, action selection, and reward [37]. The reward generated by RAT guides motor learning through dopamine secretion [38]. In the treatment of upper limb motor dysfunction in stroke patients, RAT can restore upper limb function by activating motion-related brain networks and reward circuits [17].

RAT and VRMT are both promising treatments for improving upper limb motor dysfunction in stroke patients [39]. Combined application of RAT and VRMT could theoretically activate MNS and reward circuits to a greater extent, but further high-quality research is needed before the RAVRMT robot can be widely used in clinical practice. Therefore, we design a single-center, assessor-blinded, randomized controlled clinical trial to observe if the RAVRMT robot can better improve upper limb motor dysfunction after stroke.

### Limitation

A double-blind design cannot be carried out in this study, which may cause deviations and affect the quality of the trial. However, we blind outcome assessors and statistical analysts to minimize the trial deviations.

### Trial status

Participant recruitment has not started.

## Data Availability

The datasets generated and/or analyzed after completing the current study will be available from the corresponding author by reasonable requests.

## Abbreviations

RAVRMT: Robot-assisted virtual reality mirror therapy
VRMT: Virtual reality mirror therapy
RAT: Robot-assisted therapy
fMRI: Functional magnetic resonance imaging
FMA-UE: Fugl-Meyer motor function assessment-Upper Extremity
SD: Standard deviations
MoCA: Montreal cognitive assessment
ADL: Ability of daily living
QOL: Quality of life
SF-36: Short Form 36-Item Health Survey
VAS: Visual analogue scale
IA: Internet addiction
SSQ: Simulator sickness question
MNS: mirror neuron system
